# A New RBPs-Related Signature Predicts the Prognosis of Colon Adenocarcinoma Patients

**DOI:** 10.3389/fonc.2021.627504

**Published:** 2021-03-09

**Authors:** Kaili Chang, Chong Yuan, Xueguang Liu

**Affiliations:** Department of Pathology, School of Basic Medical Sciences, Fudan University, Shanghai, China

**Keywords:** colon adenocarcinoma (COAD), RNA binding proteins (RBPs), prognostic, signature, nomograph

## Abstract

The dysregulation of RNA binding proteins (RBPs) is closely related to tumorigenesis and development. However, the role of RBPs in Colon adenocarcinoma (COAD) is still poorly understood. We downloaded COAD’s RNASeq data from the Cancer Genome Atlas (TCGA) database, screened the differently expressed RBPs in normal tissues and tumor, and constructed a protein interaction network. COAD patients were randomly divided into a training set (N = 315) and a testing set (N = 132). In the training set, univariate Cox analysis identified 12 RBPs significantly related to the prognosis of COAD. By multivariate COX analysis, we constructed a prognostic model composed of five RBPs (CELF4, LRRFIP2, NOP14, PPARGC1A, ZNF385A) based on the lowest Akaike information criterion. Each COAD patient was scored according to the model formula. Further analysis showed that compared with the low-risk group, the overall survival rate (OS) of patients in the high-risk group was significantly lower. The area under the curve of the time-dependent receiver operator characteristic (ROC) curve was 0.722 in the training group and 0.738 in the test group, which confirmed a good prediction feature. In addition, a nomogram was constructed based on clinicopathological characteristics and risk scores. C-index and calibration curve proved the accuracy in predicting the 1-, 3-, and 5-year survival rates of COAD patients. In short, we constructed a superior prognostic and diagnostic signature composed of five RBPs, which indicates new possibilities for individualized treatment of COAD patients.

## Introduction

Colorectal cancer is the third most common cancer and the third most common cause of cancer-related death (1). By 2030, global new cases of colorectal cancer infection are expected to exceed 2.2 million, and the death toll will reach 1.1 million (2). Colon adenocarcinoma (COAD) is the most common type of colorectal cancer (3) and primarily occurs in the intestinal mucosa. COAD usually grows into the intestinal lumen and spreads to adjacent organs. It is a highly aggressive malignant tumor with a high mortality and recurrence rate (4, 5). Although the clinical treatments for colon cancer, including surgical techniques, radiotherapy and chemotherapy, have been improved, the prognosis of patients is still poor (6, 7). Genes were affected by a variety of regulatory mechanisms, including but not being limited to DNA methylation, histone deacetylation and miRNA expression, thereby promoting the occurrence and development of tumors (8, 9). Therefore, discovery of new regulatory factors and therapeutic targets for COAD is imperative to improve our understanding of cancer occurrence and disease progression.

Post-transcriptional gene regulation (PTGR) is necessary in order to maintain cell metabolism and coordinate the maturation, transportation, stabilization and degradation of various types of RNA. RNA-binding proteins (RBPs) play a key role in the processes of PTGR (10). They interact with target gene mRNAs and manipulate the processing of these mRNAs to determine cell behaviors (11). In fact, each of these events is regulated by the formation of ribonucleoprotein (RNP) complex with different core RBPs (12). Recent studies have revealed that RBPs are associated with neurodegenerative diseases, muscle atrophy, diabetes, as well as different cancers and developmental disorders (13–16). However, the role of RBPs in cancer continues to be poorly understood.

RBPs play a major role in the development of colon cancer. RBM3, a member of RBPs, is up-regulated in a phase-dependent manner, and its overexpression can induce oncogenic transformation (17). Increased expression of CELF1 leads to growth arrest of intestinal epithelial cells in G1 phase, while its silence promotes cell proliferation (18). Overexpression of LIN28B is associated with an aggressive phenotype, worsened survival rate and increased recurrence of tumor (19, 20). However, these studies are still far from enough to explore the panorama of RBPs in COAD. Therefore, we obtained the expression data and patient data of COAD patients from TCGA, explored the functions of RBPs through a series of bioinformatics and statistical analysis, and constructed a prognostic signature composed of five RBPs to predict the prognosis of COAD patients. In addition, we constructed a nomogram, hoping to be able to provide valuable insights for the individualized treatment of COAD patients.

## Materials and Methods

### Data Processing

We obtained RNA sequencing data of 41 normal colon tissue samples and 473 tumor samples and corresponding data of 447 patients from the official website of the Cancer Genome Atlas Database (https://portal.gdc.cancer.gov/). M stage was removed in multivariate analysis because of too much missing information. Moreover, we obtained 1542 RBPs from these articles (10, 21). We used limma (22) package to analyze the difference of RBPs, | logFC | ≥ 0.5, FDR pvalue < 0.05 was the cut-off value.

### KEGG Pathway and GO Enrichment Analysis

We used clusterProfiler (23) package to do GO enrichment and Kyoto Genome Encyclopedia (KEGG) analysis method to comprehensively analyze the biological functions of differentially expressed RBPs. GO analysis terms include cell component (CC), molecular function (MF) and biological process (BP). FDR *P* value<0.05 as a filter condition.

### PPI Network Construction

Differentially expressed RBPs were submitted to the string database to determine the information on protein-protein interactions (https://string-db.org/). Using Cytoscape V3.8.0 software was used for visualization and the most relevant sub network modules were obtained by using the molecular complex detection (MCODE) (24) plug-in. P ≤0.05 represents significant difference.

### Construction and Verification of Prediction Model

Univariate Cox analysis was performed in the training set to screen the differences of RBPs related to overall survival (OS) in COAD patients, and then based on multivariate Cox regression analysis of the lowest Akaike information criterion (AIC) value, a proportional risk regression model of 5 RBPs prognosis signature were obtained. The risk score was calculated based on the expression of these 5 genes. The risk score formula was as follows: $\text{Risk Score}=\sum_{i=1}^{n}Coef(i)\times x(i),where\;Coef\;(i)\;and\;x(i)$ represent the regression coefficient and the expression value of each prognosis related RBPs, respectively. According to the median value, COAD patients were divided into high and low risk groups. The Kaplan-Meier survival curve was used to compare survival differences. Principal component analysis (PCA) was used to visualize gene expression patterns. The receiver-operating characteristic (ROC) curves were performed evaluate the prognostic accuracy of the model. In addition, the testing set was applied to confirm the prediction ability of the prediction model.

### Establishment and Validation of Nomogram

In the training set, we constructed a nomogram to predict the survival of COAD patients in 1, 3, 5 years by combining the clinicopathological characteristics of age, gender, stage, T stage and N stage, as well as the risk score obtained from prognostic signature. In addition, the concordance index (C-index) was used to evaluate the descriptive and predictive capabilities of nomograms. The calibration curve of nomogram was performed to test whether the predicted survival rate was consistent with the actual survival rate.

### Verification of Express Level and Prognostic Significance

The Wilcoxon test was used to detect the expression of 5 RBPs at the transcriptional level in COAD patients. Kaplan Meier survival curve proves the prognostic valued of RBPs.

### Statistical Analysis

In this study, Strawberry Perl for windows (Version5.18.2) was used for data processing, and R (3.6.2) was performed for data analysis. *P* < 0.05 was considered statistically significant.

## Result

**Figure 1:**
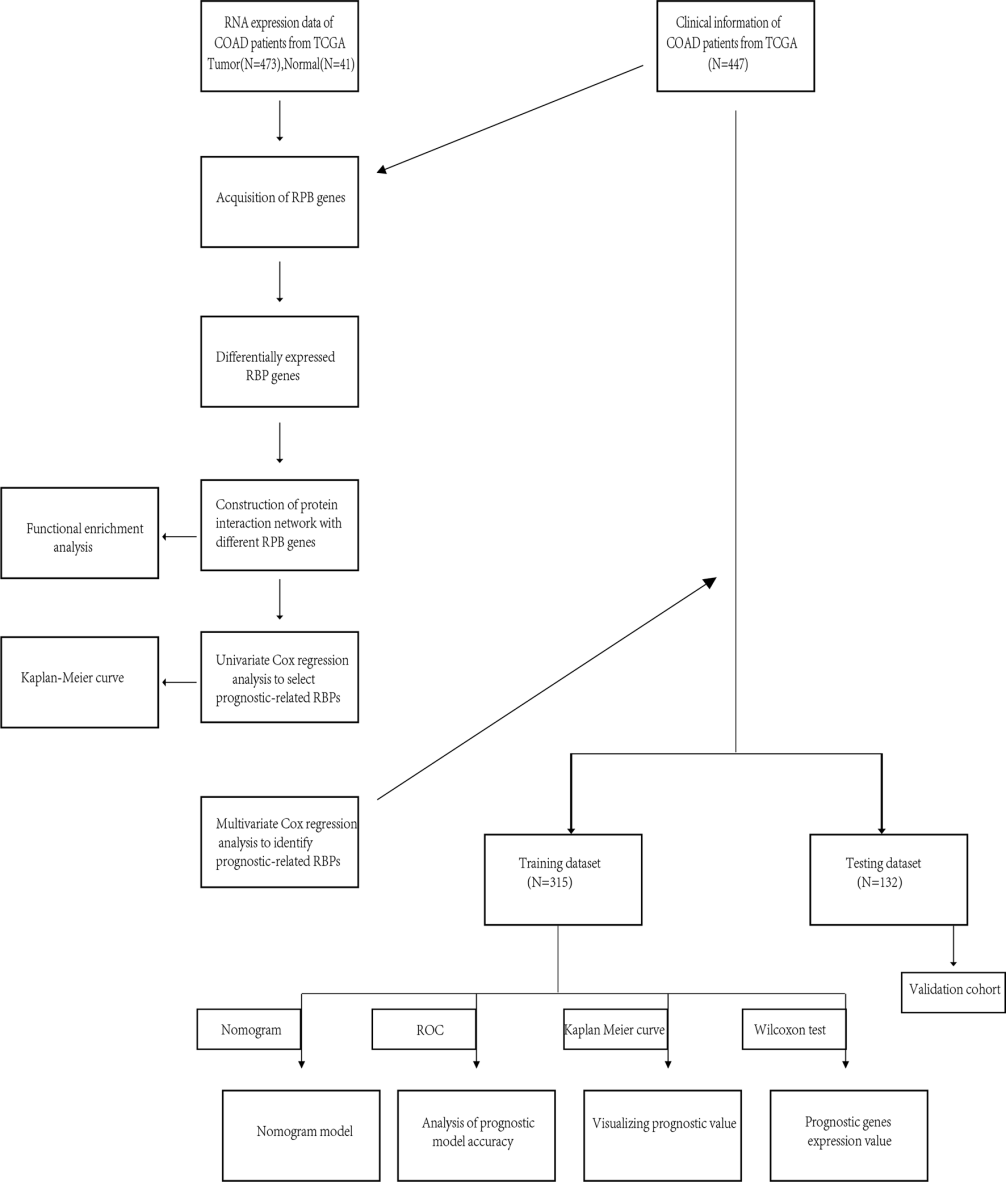
Whole procedures for analyzing RBPs in COAD.

The flow of this research was presented in **Figure 1**. A total of 447 COAD patients from the TCGA-COAD cohort were enrolled and were randomly divided into training set (N = 315) and testing set (N = 132). **Table 1** summarized the detailed clinical characteristics of these patients.

**Table 1:** Clinical characteristics of the COAD patients used in this study.

		Training datasets	Testing datasets
No. of patients		315	132
Age (median, range)		68 (35–90)	69 (31–90)
Gender (%)			
	Female	139 (44.1%)	73 (55.3%)
	Male	176 (55.9%)	59 (44.7%)
Stage (%)			
	Stage 1	54 (17.1%)	21 (15.9%)
	Stage 2	126 (40%)	50 (37.9%)
	Stage 3	81 (25.7%)	43 (32.6%)
	Stage 4	45 (14.3%)	16 (12.1%)
	Unknown	9 (2.9%)	2 (1.5%)
T stage (%)			
	T1–2	63 (20%)	23 (17.4%)
	T3–4	251 (79.7%)	109 (82.6%)
	Unknown	1 (0.3%)	
M stage (%)			
	M0	229 (72.7%)	101 (76.5%)
	M1	45 (14.3%)	16 (12.1%)
	Unknown	41 (13%)	15 (11.4%)
N stage (%)			
	N0	193 (61.3%)	773 (55.3%)
	N1–2	122 (38.7%)	59 (44.7%)
Survival status			
	Death	62 (19.7%)	34 (25.8%)
	Living	253 (80.3%)	98 (74.2%)

## Identification of Different RBPs

**Figure 2:**
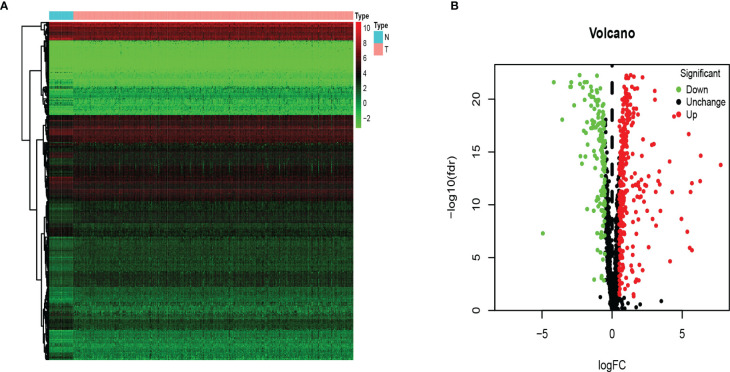
The differentially expressed RBPs in COAD. **(A)** Heat map. Columns represent tissue samples, and rows represent differentially expressed RBP. N, normal; T, tumor. **(B)** Volcano plot. The selection criteria were |logFC| > 0.5 and FDR p value < 0.05.

We analyzed RNA-seq data from 473 COAD samples and 41 normal colon tissues from the TCGA database and obtained 1,542 RBPs gene expression data. We followed (FDR *P* value < 0.05, | logFC | > 0.5) as the screening criteria to obtain 321 up-regulated genes and 151 down-regulated genes (**Figures 2A, B**).

### Functional Enrichment Analysis of Differential RBPs

**Figure 3:**
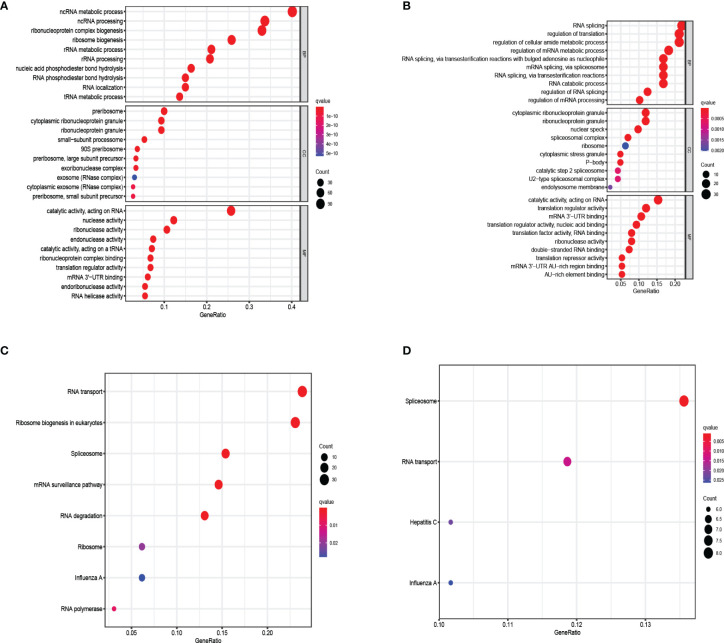
KEGG pathway and GO enrichment analysis of aberrantly expressed RBPs. **(A)** Enrichment of up-regulated RBPs in GO. **(B)** Enrichment of down-regulated RBPs in GO. **(C)** Enrichment of up-regulated RBPs in KEGG. **(D)** Enrichment of down-regulated RBPs in KEGG.

In order to explore the biological processes and functions of these differentially expressed genes, we performed gene ontology (GO) and Kyoto encyclopedia of genes and genomes (KEGG) analysis on 321 up-regulated genes and 151 down-regulated genes, respectively. The result showed that up-regulated RBPs were significantly enriched in the biological process (BP) ribosome-related processes, including ribosome biogenesis, ribonucleoprotein complex biogenesis, and rRNA metabolic process. Down-regulated RBPs were mainly enriched in RNA cleavage, including RNA fragmentation, mRNA metabolic process, and RNA splicing. In terms of cell components (CC), the up-regulated differentially expressed RBPs were mainly ribosomal components, such as 90S preribosome, small-subunit processome, and ribonucleoprotein granule. Down-regulation of RBPs mainly existed in spliceosomal complex and catalytic step 2 spliceosome. However, molecular function analysis showed that up-regulated RBPs increased significantly nuclear activity, ribonuclease activity and endonuclease activity, while down-regulated RBPs significantly decreased translation reporter activity, ribonuclease activity and mRNA 3 ‘- UTR binding (**Figures 3A, B**). Furthermore, KEGG analysis showed similar results. Up-regulated RBPs were significantly enriched in ribosome and RNA degradation (**Figure 3C**), while down-regulated PBRs were enriched in RNA transport and spliceosome (**Figure 3D**).

### Protein-Protein Interaction Network Construction

**Figure 4:**
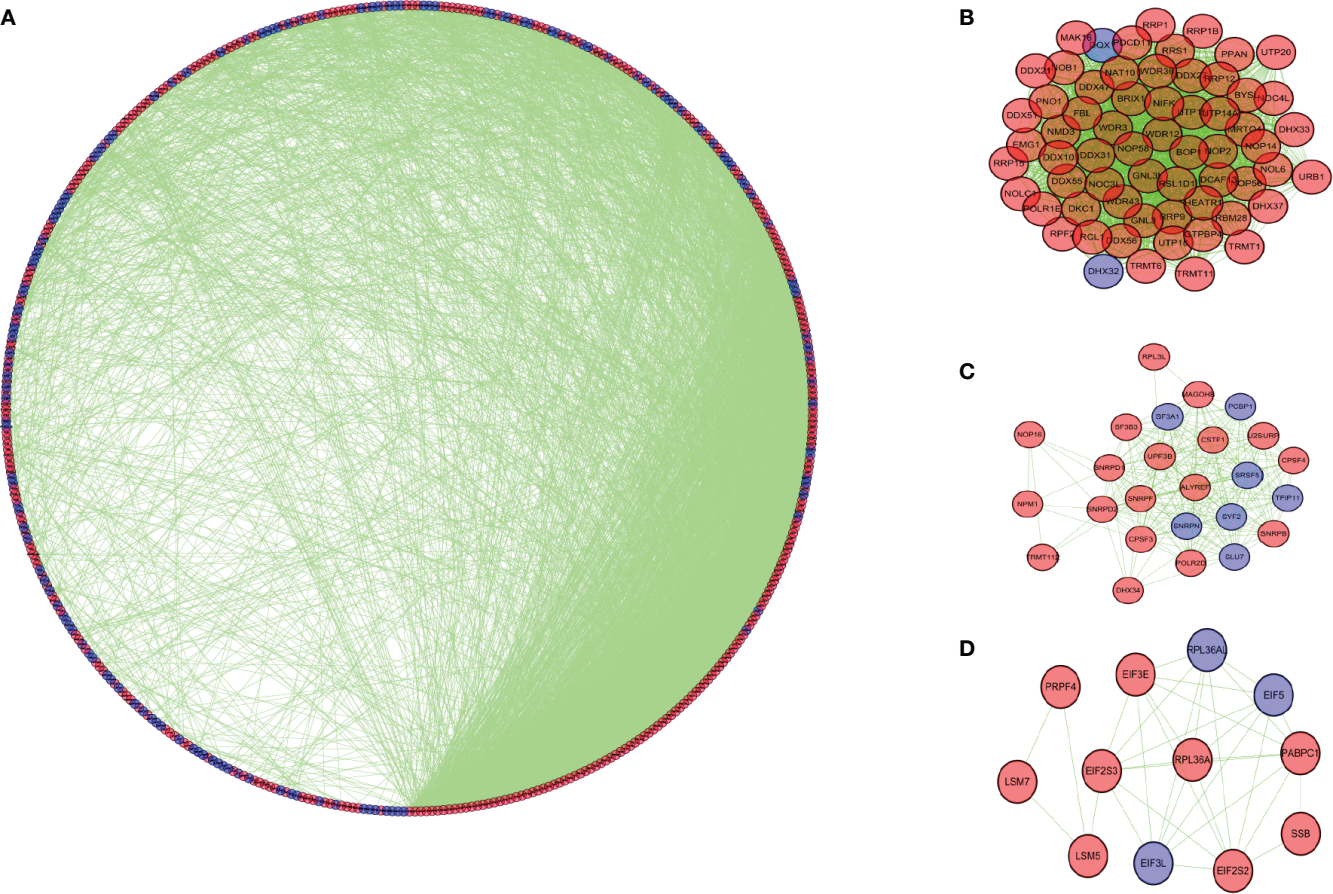
Protein-protein interaction network and modules analysis. **(A)** Protein-protein interaction network of differentially expressed RBPs. **(B)** Module 1 in PPI network. **(C)** Module 2 in PPI network. **(D)** Module 3 in PPI network. Blue circles: down-regulation; red circles: up-regulation.

We further studied the role of differentially expressed RBPs in COAD. Through the STRING database, we obtained the PPI network with required interaction score greater than 0.9, which includes 464 nodes and 2,288 edges. Cytoscape software was used for visualization (**Figure 4A**). The MCODE tool was applied to identify possible key modules. **Figures 4B** and **D** showed the three most important modules. The first important module was shown in **Figure 4B**. Ribosome biogenesis, rRNA metabolic process, and rRNA processing were significantly enriched in module 1 with 63 RBPs (**Table S1**).

### Construction and Evaluation of Prognostic Signature

**Figure 5:**
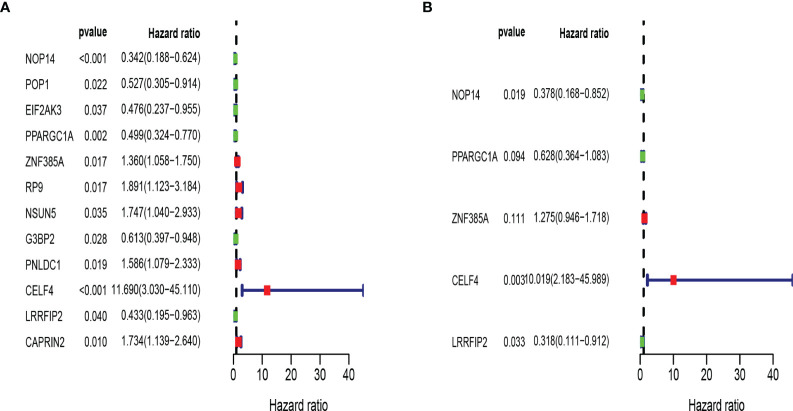
Establishment of RBPs-related prognosis model in training set. **(A)** Univariate Cox regression analysis for identification of prognosis related RBPs. **(B)** Multivariate Cox regression analysis for constructing model.

Then we constructed a prognostic model in the training set. Univariate Cox regression analysis showed that 12 RBPs (NOP14, POP1, EIF2AK3, PPARGC1A, ZNF385A, RP9, NSUN5, G3BP2, PNLDC1, CELF4, LRRFIP2, CAPRIN2) gene expression levels were significantly correlated with the overall survival rate (**Figure 5A**; *P* < 0.05). Hazard ratios (HRs) identify risk-related genes (HR > 1) and protective genes (HR < 1). Subsequently, multivariate Cox regression analysis was performed on the candidate RBPs-related genes to evaluate their roles as independent prognostic factors for patient survival. Based on the lowest Akaike information criterion (AIC) value, we identified 5 RBPs (CELF4, LRRFIP2, NOP14, PPARGC1A, ZNF385A) as prognostic signature (**Figure 5B**).

**Figure 6:**
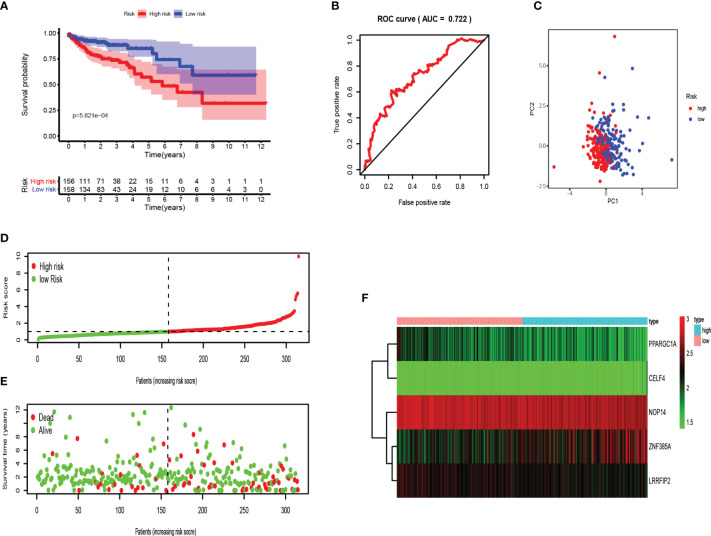
Validation of the RBPs-related prognostic signature in training set. **(A)** Kaplan-Meier survival curve showed that the survival time of the high-risk group was significantly shorter than that of the low-risk score group. **(B)** ROC curve analysis of risk score as an independent prognostic factor. **(C)** PCA showed that five RBPs are distributed differently between high-risk groups and low-risk groups. **(D)** As the risk score increases, the distribution of high and low risk COAD patients. **(E)** The scatter plot displayed the correlation between the survival status of high- and low-risk COAD patients. **(F)** The heat map showed the differences in the expression of five RBPs between high and low risk patients.

The risk score of each patient was determined according to the prognostic signature model, and COAD patients were divided into high and low-risk groups in the light of the median value of risk value. Kaplan Meier survival curve showed that the survival rate of COAD patients in high-risk group was significantly lower than that in low-risk group (**Figure 6A**). Receiver operating characteristic (ROC) curve was performed to evaluate the prognosis of the model (**Figure 6B**). The area under the curve (AUC) was 0.722 which indicated good prediction ability. Principal component analysis showed that patients with high and low risk had different distribution patterns (**Figure 6C**). We ranked patients from low to high according to risk value, and the scatter plot demonstrated the survival rate of high-risk patients was much lower than that of low-risk patients (**Figures 6D, E**). The heat map displayed the difference of 5 RBPs genes expressions in high and low risk groups (**Figure 6F**).

**Figure 7:**
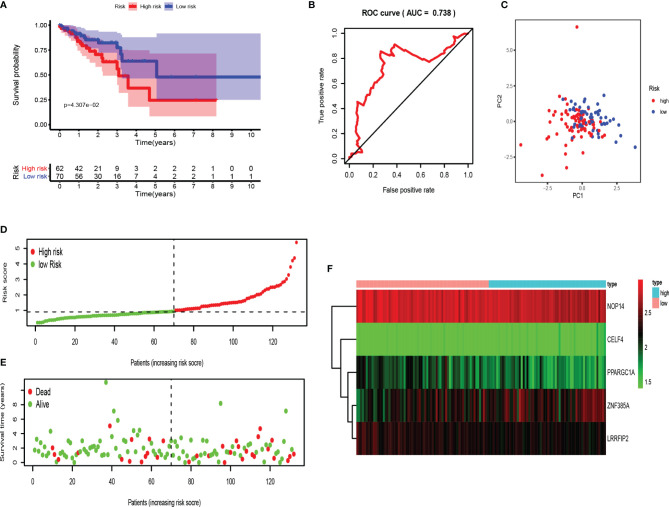
Evaluation of the RBPs-related prognostic signature in the testing set. **(A)** Survival curve for low- and high-risk subgroups. **(B)** ROC curves for forecasting OS based on risk score. **(C)** Distribution pattern of PCA in different risk subgroups. **(D)** Risk score distribution of COAD patients. **(E)** Survival status of COAD patients. **(F)** Expression heat map.

Moreover, testing set was applied for external verification. As shown in **Figures 7A–C**, the survival rate of patients in the high-risk group was lower than that in the low-risk group. The area under ROC was 0.738, and PCA analysis showed different distribution. These data proved that RBPs-related genes can accurately predict the survival of COAD patients. Similarly, we analyzed the risk characteristics of the training set (**Figures 7D–F**). As we assumed, the risk signatures of the five RBPs-related genes were robust.

### RBPs-Related Gene Prognostic Signature Is an Independent Prognostic Factor

**Figure 8:**
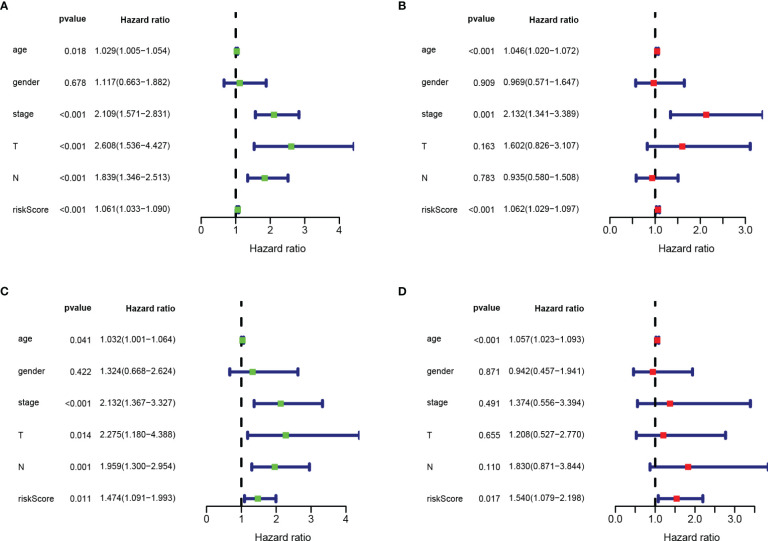
Risk score based on RBPs-related prognostic signature was an independent predictor for COAD patients. **(A)** Univariate Cox regression analysis in training set. **(B)** Multivariate Cox regression analysis in training set. **(C)** Univariate Cox regression analysis in testing set. **(D)** Multivariate Cox regression analysis in testing set.

Next, we performed univariate and multivariate Cox regression analysis based on clinicopathological characteristics (age, gender, stage, T stage, N stage) and risk scores of COAD patients, to determine whether RBPs-related gene prognostic characteristics are independent predictors for COAD patients. Univariate analysis showed that age (*P* < 0.018), stage (*P* < 0.001), T stage (*P* < 0.001), N stage (*P* < 0.001) and risk score (*P* < 0.001) were significantly correlated with OS in both training and testing sets (**Figures 8A, C**). Multivariate analysis showed that only age and risk score had significant correlation with OS in COAD patients (*P* < 0.05; **Figures 8B, D**). These data suggest that RBPs-related gene prognostic signatures are independent factors affecting the prognosis of COAD patients.

### Construction of a Nomogram

**Figure 9:**
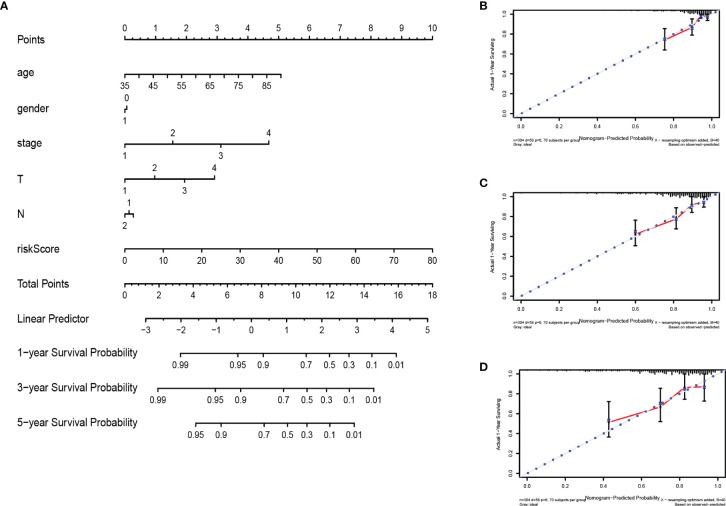
Construction and verification of nomogram. **(A)** The prognostic nomogram constructed based on the risk score of RBPs and clinicopathological parameters predicted the survival rate of COAD patients at 1, 3, and 5 years. **(B–D)** Calibration curves showed the concordance between predicted and observed 1-, 3-, and 5-year survival rates.

The nomogram integrates multiple prognostic factors to evaluate the survival probability of an individual at a specific time point and displays it graphically (25). To further predict the survival of COAD patients, we constructed a nomogram consisting of clinicopathological features (age, gender, stage, T stage, N stage) and risk score (**Figure 9A**). Nomography predicted the 1-, 3-, 5-year survival rate of COAD patients. The calibration curve showed that the actual patient survival was consistent with the predicted value (**Figures 9B–D**). The concordance index (C index) of nomogram was 0.770, which proves the accurate prediction performance of the nomogram. These results indicate that the nomogram with risk score can accurately predict the 1-, 3-, 5-year survival rate of patients, and provide valuable insights for individualized treatment of COAD patients.

### Validation the Prognostic Value and Expression of RBPs

**Figure 10:**
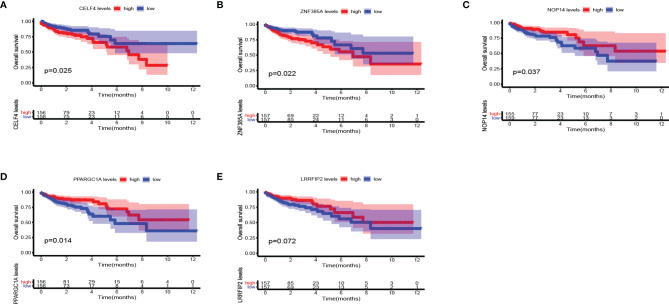
Kaplan Meier survival curve analysis verified the prognostic value of RBPs in COAD. **(A)** CELF4; **(B)** ZNF385A; **(C)** NOP14; **(D)** PPARGC1A; **(E)** LRRFIP2.

**Figure 11:**
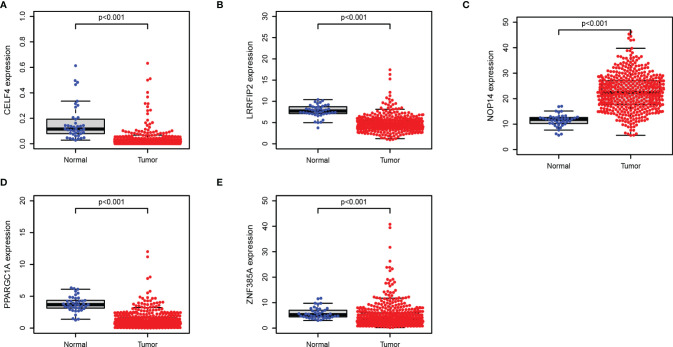
Verification of the transcription expression of RBPs in COAD and normal colon tissues. **(A)** CELF4; **(B)** LRRFIP2; **(C)** NOP14; **(D)** PPARGC1A; **(E)** ZNF385A.

We further explored the prognostic value and expression value of five RBPs in COAD patients. The Kaplan-Meier survival curve showed that the expressions of CELF4, NOP14, PPARGC1A, and ZNF385A were correlated with the survival of COAD patients (all *P* < 0.05; **Figures 10A–D**), while the remaining LRRFIP2 was not significantly correlated with survival (*P* = 0.072; **Figure 10E**). We then analyzed whether there were differences in the expressions of these genes between normal and cancer tissues. Surprisingly, NOP14 was up-regulated in tumors and the other four (CELF4, PPARGC1A, ZFF385A, LRRFIP2) were down-regulated in tumors (**Figure 11**).

## Conclusion and Discussion

RNA-binding proteins (RBPs) play an important role in cellular homeostasis by controlling gene expression at the post-transcriptional level (26). RBPs are dysregulated in several cancer types, affecting the expressions and functions of both tumor proteins and tumor-suppressor proteins (27). In this study, we obtained the original RNASeq data of COAD patients from TCGA and identified 472 differentially expressed RBPs genes. Then we performed enrichment analysis on up-regulated genes and down-regulated genes. The results showed that the up-regulated RBPs were significantly enriched in the ribosome-related processes, including ribosome biosynthesis, ribonucleoprotein complex biosynthesis, and rRNA metabolism. Down-regulated RBPs were mainly enriched in RNA cleavage, including RNA fragmentation, mRNA metabolic process, and RNA splicing. Relevant studies have proved that the regulation of RNA processing and RNA metabolism is related to the development of COAD (28, 29). In addition, we constructed a PPI network to comprehensively display the correlation between differentially expressed RBPs proteins in COAD.

RBPs are a group of genes that regulate the growth, development and survival of cancer cells, and are closely related to cancer progression (30, 31). For instance, IGF2BP3 may contributes to lung tumorigenesis by regulating the alternative splicing of PKM (32), HuR promotes the progression of head and neck squamous cell carcinoma and bladder cancer (33, 34). Therefore, RBPs are potential biomarkers that most likely predict cancer risk and survival outcomes. In this study, we systematically analyzed the prediction accuracy of the prognostic signature of RBPs in COAD using bioinformatics and statistical tools. We randomly divided COAD patients into training set (N = 315) and testing set (N = 132) at 7:3. The multivariate COX regression analysis identified 5 genes as the optimal items for constructing a prognostic model based on the lowest AIC value. We verified the accuracy of RBPs in predicting the prognosis of COAD patients both in the training set and testing set. Furthermore, multivariate COX analysis confirmed that the risk score based on the prognostic signature of RBPs was an independent predictor of COAD patients (*P* < 0.05). Nomography is widely used to predict the survival rate of cancer patients (35). We constructed and evaluated a nomogram composed of clinicopathological characteristics (age, gender, stage, T stage, N stage) and risk scores. These results suggest that the present RBPs prognostic signature accurately predicts the survival of COAD patients.

Recently, a Cox model based on seven RBPs was reported to predict the survival of patients with COAD, two of which (PPARGC1A and LRRFIP2) were also present in our model (36). Among the five RBPs in our model, the other three genes (CELF4, NOP14, ZNF385A) were not previously reported to be associated with COAD. We used the Kaplan-Meier survival curve to explore the prognostic value of these five genes. CELF4, LRRFIP2, NOP14 and ZNF385A were associated with OS in COAD patients (*P* < 0.05), while PPARGC1A was not significantly correlated with the overall survival of COAD patients (*P* = 0.072). However, since we applied the average gene expression value instead of the best P value as the cutoff criteria to divide the high and low expression groups, we cannot completely deny the prognostic value of PPARGC1A.

RBPs regulate the expressions of genes required for many aspects of cancer behaviors including its sensitivity to chemotherapy in post-transcriptional network (37). In the identified five RBPs, the expressions of CELF4, LRRFIP2, ZNF385A, and PPARGC1A were down-regulated, while the expression of NOP14 was up-regulated (all *P* < 0.001). As we know, the main treatment of colon cancer is surgical resection, supplemented by chemotherapy and radiotherapy. Chemoresistance in colorectal cancer is urged to be conquered. CELF4 (CUGBP, ELAV-like family member 4) is one of six mammalian CELF proteins that function in mRNA metabolism. Current studies have shown that CELF4 genetic variation contributes to chemotherapy-related cardiac dysfunction (38, 39). Whether CELF4 contributes to chemoresistance in COAD patients is not reported yet. LRRFIP2 (Leucine-rich repeat flightless-interacting protein 2) is a signal regulator that interacts with Toll-like receptor (TLR) adaptor protein MyD88 to regulate NF-κB activity (40). NF-κB activates the expression of c-MYC, ICAM-1, and VEGFA, and promotes the growth and proliferation of colon cancer cells (41). This suggests that targeting LRRFIP2 may be effective for COAD treatment. ZNF385A (Zinc finger protein 385A) acts as a transcription factor which modulates the activation of PAK-2p34 by proteasome-mediated degradation. ZNF385A is related to cognitive decline in one’s later years (42), however, its relationship with cancer is rarely mentioned. Since we found that the high level of ZNF385A indicates the poor prognosis of COAD patients, it may be a potential target for further COAD research. NOP14 (NOP14 Nucleolar Protein) plays a role in pre-18s rRNA processing and small ribosomal subunit assembly. It is associated with a variety of cancer progression including pancreatic cancer (43), melanoma (44) and breast cancer (45). Although NOP14 lacks detailed research in the field of COAD, we speculate that it has the ability to promote tumor progression based on the literature. Thus, we screened out five RBPs which have a huge excavation potential and provide probable targets for COAD treatment.

In conclusion, we constructed a prognostic signature of RBPs which can accurately predict the survival outcome of COAD patients. We combined the prognostic signature and other clinicopathological characteristics to establish and verify a prognostic nomogram. Our data indicates that the identified five RBPs are the potential prognostic and diagnostic biomarkers, which provide a valuable reference for practitioners’ clinical decision and enable individualization of COAD treatment.

## Data Availability Statement

The original contributions presented in the study are included in the article/**Supplementary Material**. Further inquiries can be directed to the corresponding author.

## Author Contributions

CY performed the statistical analyses and drafted the manuscript. KC supervised the statistical analyses, participated in data analysis and interpretation, and provided critical feedback. KC and CY provided data for fine-mapping. All authors contributed to the article and approved the submitted version.

## Conflict of Interest

The authors declare that the research was conducted in the absence of any commercial or financial relationships that could be construed as a potential conflict of interest.
