# Supplementary material for: A New RBPs-Related Signature Predicts the Prognosis of Colon Adenocarcinoma Patients
Source: Front Oncol. 2021 Mar 9;11:627504. doi: 10.3389/fonc.2021.627504 (PMC7985171; doi:10.3389/fonc.2021.627504)
Supplement: Supplementary file 1 [file Table_1.docx]

**Table S1**

Go enrichment analysis results of genes in module 1.

| ID | Description | GeneRatio | pvalue | p.adjust | Count |
| --- | --- | --- | --- | --- | --- |
| GO:0042254 | ribosome biogenesis | 48/58 | 4.1e-78 | 1.3e-75 | 48 |
| GO:0016072 | rRNA metabolic process | 44/58 | 3.7e-74 | 6.1e-72 | 44 |
| GO:0006364 | rRNA processing | 43/58 | 1.0e-72 | 1.1e-70 | 43 |
| GO:0022613 | ribonucleoprotein complex biogenesis | 48/58 | 5.2e-68 | 4.3-66 | 48 |
| GO:0034470 | ncRNA processing | 46/58 | 1.1e-67 | 7.6e-66 | 46 |
| GO:0034660 | ncRNA metabolic process | 47/58 | 2.0e-65 | 1.1e-63 | 47 |
